# Discordant Post-natal Patterns in Fetuses With Heterotaxy Syndrome: A Retrospective Single-Centre Series on Outcome After Fetal Diagnosis

**DOI:** 10.3389/fped.2022.908505

**Published:** 2022-07-14

**Authors:** Elisabeth Seidl-Mlczoch, Gregor Kasprian, Erwin Kitzmueller, Daniel Zimpfer, Irene Steiner, Victoria Jowett, Marlene Stuempflen, Alice Wielandner, Barbara Ulm, Ina Michel-Behnke

**Affiliations:** ^1^Department of Pediatrics and Adolescent Medicine, Division of Pediatric Cardiology, Pediatric Heart Center, Medical University of Vienna, Vienna, Austria; ^2^Department of Biomedical Imaging and Image-Guided Therapy, Division of Neuroradiology and Musculoskeletal Radiology, Medical University of Vienna, Vienna, Austria; ^3^Department of Cardiac Surgery, Pediatric Heart Center Vienna, Medical University of Vienna, Vienna, Austria; ^4^Section for Medical Statistics, Center for Medical Statistics, Informatics and Intelligent Systems, Medical University of Vienna, Vienna, Austria; ^5^Department of Fetal Cardiology, Great Ormond Street Hospital, London, United Kingdom; ^6^Department of Obstetrics and Gynaecology, Division of Obstetrics and Fetomaternal Medicine, Medical University of Vienna, Vienna, Austria

**Keywords:** heterotaxy, isomerism, discordant pattern, foetal echocardiography, cardiac surgery, outcome

## Abstract

**Objective:**

Cardiac and extra-cardiac anomalies in 46 pre-natally diagnosed cases of heterotaxy were compared to post-natal anatomical patterns in order to reveal discordant findings. Second, the outcome of these fetuses was evaluated.

**Methods:**

Fetuses with heterotaxy, diagnosed in a tertiary referral centre, were analysed retrospectively. Based on the foetal abdominal situs view, right atrial isomerism (RAI) and left atrial isomerism (LAI) were defined as foetal sub-types. Post-natally, discordant anatomical patterns for broncho-pulmonary branching, atrial appendage morphology, and splenic status were further clarified with CT scans. In summary, the spectrum of pre-natally and post-natally detected cardiac and extra-cardiac anomalies is systematically reviewed. Necessary surgical interventions and mid-long-term outcomes were compared between the two sub-types in surviving infants.

**Results:**

A total of 46 fetuses with heterotaxy were included; LAI was diagnosed in 29 (63%) fetuses and RAI was diagnosed in 17 (37%) fetuses. Extra-cardiac anomalies were noted in 35% of fetuses. Seven out of the 29 fetuses (24%) with LAI had atrio-ventricular block (AVB) and four of these cases presented with hydrops. Twenty nine out of the 46 participating fetuses (63%) were live births, with 62% in the LAI group and 65% in the RAI group. Five fetuses were lost to follow-up. At the age of 1 year, the overall survival of live births [estimate (95% CI)] was 67% (48; 92%) in patients with LAI and 55% (32; 94%) in patients with RAI. At the age of 5 years, the estimates were 67% (48; 92%) in the LAI group and 46% (24–87%) in the RAI group. The median survival (first quartile; third quartile) was 11.1 (0.1; 14) years for patients with LAI and 1.3 (0.09; NA) years for patients with RAI. Of 17 children who had undergone cardiac surgery, five (29%) children achieved a bi-ventricular repair and 12 (70%) children achieved a uni-ventricular palliation. Three were primarily palliated, but converted to bi-ventricular thereafter. Foetal subtype definition of heterotaxy based on the abdominal situs and post-natal thoracic imaging studies showed a discordant pattern of broncho-pulmonary branching and atrial appendage anatomy in 40% of our live-born children.

**Conclusion:**

Heterotaxy is a rare and complex condition with significant morbidity and mortality related to severe cardiac and extra-cardiac associations. Accurate pre-natal diagnosis can help identify the fetuses at risk and allow for timely intervention in a multi-disciplinary setting. Further studies are warranted to shed light on the exact sub-type definition in fetuses with heterotaxy and the presence of discordant post-natal patterns.

## Introduction

Heterotaxy syndrome (HS) is a rare congenital disorder with an incidence of 1 in 5,000–7,000 live births (1), characterised by an abnormal arrangement of structures relative to the left-right axis of the foetal body (2, 3), including the atrial appendages and the internal thoraco-abdominal organs. The left and right atria can be identified by the morphology of the atrial appendage, which has a specific pattern of pectinate muscles, referring to a right atrium and a left atrium. The normal arrangement, with asymmetrically arranged thoracic and abdominal organs, is called “situs solitus.” Both the nomenclature and classification are a matter of discussion. As a result, the International Society for Nomenclature of Paediatric and Congenital Heart Disease proposed a definition in 2007 (4) to facilitate the precise use of the term, which will be used in this study. The definition of HS usually implies the description of two groups based on the morphology of the symmetrical side: left atrial isomerism (LAI) and right atrial isomerism (RAI). Historically, LAI and RAI have been distinguished based on certain features such as the presence or absence of a spleen (5). However, while there seems to be no pathognomonic pattern in each group, certain associated patterns have been described to occur more often in the respective groups (2, 4, 6).

In LAI, there are usually two morphological left atrial appendages, bilateral bi-lobed lungs, long bronchi on both the sides, and a situs anomaly of the abdominal organs, typically along with balanced congenital heart defects (CHDs), an interrupted inferior vena cava (IVC), and non-cardiac conditions such as malrotation of the intestines and polysplenia and biliary atresia (6).

In RAI, two morphological right atrial appendages can be found, as well as bilateral tri-lobed lungs, two short bronchi on both the sides, together with typically severely unbalanced CHD involving abnormal drainage of the pulmonary veins and abnormal intra-abdominal location of the aorta and the IVC; asplenia is also found in the majority of cases (6). Most of the post-natal features defining the HS subtypes (RAI vs. LAI), such as broncho-pulmonary branching, atrial appendage arrangement, and splenic status, are difficult to visualise in foetal scanning. To visualise the normal abdominal situs arrangement, a cross-sectional view of the foetal abdomen is obtained. In situs solitus, the aorta and the stomach bubble can be seen to the left of the spine, while the IVC is anterior and to the right (7). Deviations from this arrangement raise the suspicion of HS (8). Identification of the subtype in the foetus relies mainly on the 184 position of the IVC and the aorta in the abdominal situs view (8, 9). Interruption of IVC is seen in the majority of cases with LAI, whereas a juxtaposition of the IVC and the aorta is present in the majority of cases with RAI (8, 10). The thoracic situs is usually concordant with the abdominal situs, but cases with discordance have been described (4). Additional observations may point to a particular subtype. For example, the presence of total anomalous pulmonary venous drainage (TAPVD) is more likely in RAI (11). In LAI, the intra-cardiac anatomy can be normal, but this is very rare in right isomerism. Recent studies have shown that the currently used foetal HS definition is not always congruent with post-natal findings (12–14). Yim et al. reported a discordance of broncho-pulmonary branching, atrial appendage arrangement, and splenic status in more than one-fifth of patients with HS (13). Houyel et al. found non-classic patterns in 27 (44%) out of 61 foetal specimens (14). Accurate foetal diagnosis of included anomalies is crucial for counselling families about post-natal inter-disciplinary management. Ascertainment of the cardiac defect is important, as the post-natal prognosis is largely determined by the cardiac course (15–17). However, associated non-cardiac anomalies, especially anomalies of the mid-line spectrum (18), also influence morbidity and mortality. This is supported by recently published data on foetal MRI in the same HS cohort (19). Children with HS often exhibit a complex peri-natal and long-term course with significant morbidity and mortality (8–11, 20–24). However, recent studies report better outcomes, due to improved post-natal care (1, 17, 24, 25).

This study aimed to: (1) assess post-natal concordance or discordance of the sub-type of HS (LAI vs. RAI), (2) describe the spectrum of associated cardiac, non-cardiac, and chromosomal anomalies, and (3) examine pre- and peri-natal mortality and morbidity, as well as long-term outcomes in a cohort of live-born children with pre-natal diagnosis of HS in a single tertiary referral centre.

## Materials and Methods

### Study Population and Design

This is a retrospective cohort study of foetal and paediatric patients collected from the databases of the Department for Obstetrics and Gynaecology, Division of Paediatric Cardiology, and Department of Paediatric and Adolescent Medicine of the Medical University of Vienna. The ethical committee of the Medical University of Vienna approved the study protocol (1306/2020). Fetuses diagnosed with HS and a cardiac malformation between January 1998 and December 2019 were included. The pre-natal diagnoses were made with a variety of imaging investigations such as detailed foetal echocardiography (FE), obstetric ultrasound (US), and foetal MRI according to the published guidelines (7, 26, 27). The foetal MRI results of 27 fetuses of the 46 fetuses have been already reported (19). Post-natal verification was feasible with autopsy reports, post-mortem MRI (pm MRI), US, echocardiography, and CT.

Clinical data on the foetal and post-natal course (including time of interventions/surgery) and outcome at last follow-up were obtained from patients’ records, echocardiography reports, imaging reports, and autopsy reports. Fetuses diagnosed with HS whose subgroup could not be determined were excluded from the study. Type of HS was diagnosed with FE on the abdominal situs view: the presence of an interrupted IVC with azygos continuity was found to most likely suggest LAI and juxtaposition of the aorta and IVC in combination with a cardiac malformation and abnormal cardiac or abdominal organ situs most likely suggested RAI (8, 9, 11). Complete atrio-ventricular block (AVB) was defined as atrial rates faster than ventricular rates with dissociation between the two. Hydrops was diagnosed when abnormal fluid accumulations were present in more than two compartments of the foetal body. Foetal cardiac defects were defined based on the anatomy and haemodynamic circulation (uni-ventricular/bi-ventricular). In the year 2009, a new leadership team in paediatric cardiology and paediatric heart surgery started a new era of care at the paediatric heart centre in Vienna. Thus, two time periods regarding long-term outcomes were separately evaluated (1998–2009 vs. 2010–2021). Available CT scans of live-born children and available post-mortem MRI in terminated cases were evaluated regarding concordance or discordance of broncho-pulmonary branching and atrial appendage arrangement. The splenic status was assessed with the post-natal US. Broncho-pulmonary branching was defined as situs solitus, situs inversus, LAI, and RAI. LAI was defined as the pulmonary artery coursing backwards over the upper lobar bronchus (epbronchial pulmonary artery-hyparterial bronchus) (13, 28). RAI was defined as the descending branch of the pulmonary artery coursing backwards below the upper lobar bronchus (hypo-bronchial pulmonary artery-eparterial bronchus) (13, 28). Atrial appendage arrangement was defined as two morphological left appendages in LAI and two morphological right appendages in RAI using the pectinate muscles and shapes as landmarks (29, 30). The splenic status was described in post-natal US either by the morphology (regular, abnormal shaped) or by the number (polysplenia or asplenia) (13).

### Statistical Analysis

Demographic, anatomic, procedural, and outcome data were included in the analysis. Qualitative variables are summarised as frequencies (percentage) and metric variables are summarised as medians (range), if not stated otherwise. The uni-variate Cox regression models (R-package survival 3.2-10) were applied with the survival time in years as a dependent variable. An estimate for the hazard ratio (HR) with 95% confidence limits and the *p*-value (H0:HR = 1) is reported. The Kaplan–Meier curves were plotted for LAI and RAI separately. Median survival (first quartile Q1; third quartile Q3) is reported, as well as the Kaplan–Meier estimates with 95% confidence limits at different time points, respectively. Statistical analysis was performed with R 4.0.5. A value of *P* < 0.05 was considered statistically significant. Due to the explorative character of the study, we did not adjust for multiple testing. The interpretation of the *p*-values is descriptive.

## Results

### Prenatal Cohort

**FIGURE 1:**
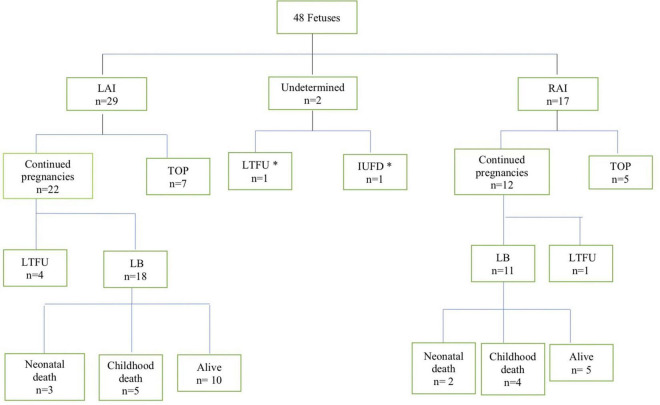
Flowchart of patients with the pre-natal diagnosis of heterotaxy in this cohort. LAI, left atrial isomerism; RAI, right atrial isomerism; TOP, termination of pregnancy; LTFU, lost to follow up; IUFD, intrauterine fetal demise; LB, livebirth. *These two fetuses were excluded from further analysis.

During the study period, 48 fetuses met the inclusion criteria of prenatally diagnosed HS. The flowchart of the study population is shown in Figure 1. Two fetuses were excluded from further analysis (classification of LAI or RAI could not be established). A total of 46 fetuses (29 LAI, 63%; 17 RAI, 37%) were included for in-depth evaluation. The clinical characteristics of the study population are shown in Table 1. Termination of pregnancy (TOP) only occurred from 2003 to 2018 in 7/29 (24%) fetuses with LAI and 5/17 (29%) fetuses with RAI. For ongoing pregnancies, no intrauterine death occurred in the cohort. In total, five fetuses were lost to follow-up during pregnancy with no available outcome data.

**TABLE 1:** Clinical characteristics of the study population.

No of patients included	46
Maternal age at diagnosis (mean)	30 (Range 17–42) years
Gestational age at diagnosis (mean)	23 (Range 12–36) weeks
Gender known	40/46
Male sex	23/40 (58%)
Left atrial isomerism (LAI)	29/46 (63%)
Right atrial isomerism (RAI)	17 (38%)
**Genetic assessment**	**31/46 (67%)**
Karyotype	27 (58%)
Karyotype including microarray	1
Non-invasive prenatal testing (Trisomies 21, 18, 13)	1
Next generation sequencing	1
Postnatal chromosomal investigation	1

### Cardiac Malformations

A detailed description of the summary of foetal and postnatal cardiac findings is shown in Table 2. Abnormal atrio-ventricular connection or significant hypoplasia of the left or right ventricle was present in 10 out of 29 (10/29, 34%) fetuses with LAI and in 13/17 (76%) fetuses with RAI and a complete atrio-ventricular septal defect (AVSD) was diagnosed in 9/29 (31%) fetuses with LAI and in 10/17 (59%) fetuses with RAI. Anomalies of the pulmonary venous drainage were present in 2/29 (7%) fetuses with LAI and 9/17 (53%) fetuses with RAI. Nine fetuses (9/46, 20%; all with LAI) had no intra-cardiac defect. 20/29 (69%) fetuses with LAI were expected to require open-heart surgery due to the complex cardiac defect in the first year of life. Foetal arrhythmia was only identified in the LAI group: 7/29 (24%) fetuses with LAI had a higher degree of AV block.

**TABLE 2:** Distribution of cardiovascular malformations among fetuses with left atrial isomerism (LAI) and right atrial isomerism (RAI).

	LAI (*n* = 29)	RAI (*n* = 17)
**Systemic venous anomalies**		
Interrupted inferior vena cava	18 (62)	1 (6)
Bilateral superior vena cava	9 (31)	5 (29)
Single left superior vena cava	0	1 (6)
**Pulmonary venous anomalies**		
Total anomalous pulmonary venous connection	2 (7)	9 (53)
Partial anomalous pulmonary venous connection	5 (18)	3 (18)
**Septation defects**		
Isolated ventricular septal defect	1 (4)	1 (6)
Common atrium	1 (4)	4 (23)
Complete atrio-ventricular septal defect	9 (31)	10 (59)
**Anomalies of the ventricles**		
Hypoplastic left ventricle/single right ventricle	3 (11)	3 (18)
Hypoplastic right ventricle/single left ventricle	3 (11)	4 (23)
Single ventricle morphology	4 (14)	6 (35)
**Ventriculo-arterial connection anomalies**		
Transposition/malposition of the great arteries	6 (21)	9 (53)
Double-outlet right ventricle	5 (18)	6 (35)
Double-outlet left ventricle	2 (7)	0
Truncus arteriosus communis	0	1 (6)
**Outflow tract obstructions**		
Pulmonary stenosis/atresia	8 (28)	14 (82)
Aortic stenosis/atresia	4 (14)	3 (18)
Coarctation of the aorta	8 (28)	1 (6)

*Data are given as number (percent). Some fetuses had more than one cardiac anomaly.*

### Extra-Cardiac Malformations

**FIGURE 2:**
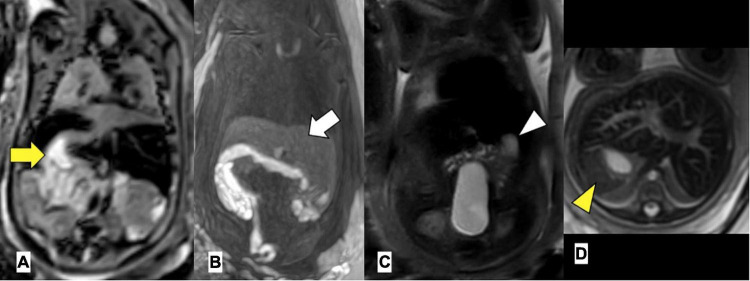
Left atrial isomerism by foetal MRI. **(A)** Arrow yellow: right-sided stomach **(B)** arrow white: bilateral liver, **(C)** arrowhead white: left-sided gallbladder, **(D)** arrowhead yellow: left-sided spleen. Gestational age at MRI is 20 weeks + 0 day.

Extra-cardiac anomalies are given in Table 3. Anomalies of abdominal organ locations were present in all the cases. An example of a foetal MRI image is shown in Figure 2. There were 7/29 (24%) fetuses with LAI who had asplenia, 13/29 (45%) fetuses had polysplenia, and 5/29 (17%) fetuses had a regular spleen. Of the fetuses with RAI 12/17 (71%) had asplenia and 4/17 (24%) had a regular left-sided spleen There were no polysplenia cases in our fetuses with RAI. In 4/46 (9%) fetuses, it was not possible to determine the presence or absence of a spleen (3 LAI, 1 RAI). Extra-cardiac anomalies (apart from the situs and spleen) were present in 16/46 (35%) of the fetuses. Twenty fetuses (10/29, 34% LAI; 10/17, 59% RAI) had abnormal findings in the central nervous system and cranio-facial anomalies. Gastro-intestinal anomalies were present in 21/46 (46%) fetuses (17/29, 59% LAI; 4/17, 24% RAI). Malrotation of the intestines was noted in 9/29 (31%) LAI cases and 3/17 (18%) RAI cases. Anomalies of the urinary tract were seen in 10/46 (22%) cases. Verification of pre-natal non-cardiac findings (imaging or autopsy) was possible in 39/46 (85%) fetuses. Pre-natal imaging findings were confirmed in 29/39 (74%) and post-natal imaging revealed additional non-cardiac malformations not previously detected on pre-natal US or MRI in 10/39 (26%) cases.

**TABLE 3:** Non-cardiac abnormalities among 46 fetuses with left atrial isomerism (LAI) (*n* = 29) and right atrial isomerism (RAI) (*n* = 17) from pre- and post-natal imaging studies and autopsy reports.

	Total number (%)	LAI number (%)	RAI number (%)
**Situs anomalies**			
Situs anomaly/ambiguus	37 (80)	25 (86)	12 (71)
Situs inversus	8 (17)	4 (14)	4 (24)
Partial situs inversus	1 (2)	0	1 (6)
**Spleen**			
Asplenia	17 (37)	7 (24)	12 (71)
Polysplenia	13 (28)	13 (45)	0
Regular spleen (right or left)	9 (19)	5 (17)	4 (24)
Small spleen	1 (2)	1 (3)	0
**Central nervous system, face**			
Craniofacial dysmorphism	6 (13)	5 (17)	1 (6)
Cleft (lip) palate	3 (6)	2 (7)	1 (6)
Ventriculomegaly	2 (4)	1 (3)	1 (6)
Ventricular asymmetry	2 (4)	1 (3)	1 (6)
Hydrocephalus	2 (4)	0	2 (12)
Cerebellar hypoplasia	1 (2)	0	1 (6)
Stenosis of the aqueduct	1 (2)	0	1 (6)
Delayed myelinisation	1 (2)	0	1 (6)
Dandy walker malformation	1 (2)	1 (3)	0
Rhombencephalosynapsis	1 (2)	0	1 (6)
**Gastrointestinal tract**			
Malrotation of the gut	12 (26)	9 (31)	3 (18)
Gallbladder aplasia	3 (6)	3 (10)	0
Duodenal atresia	2 (4)	1 (3)	1 (6)
Anal atresia	1 (2)	1 (3)	0
Omphalocele	1 (2)	1 (3)	0
Volvulus	1 (2)	1 (3)	0
Biliary atresia	1 (2)	1 (3)	0
**Urinary tract**			
Hydronephrosis	3 (6)	1 (3)	2 (11)
Duplex kidney	2 (4)	1 (3)	1 (6)
Urethral obstruction	1 (2)	1 (3)	0
Unilateral kidney agenesis	1 (2)	1 (3)	0
Bilateral kidney agenesis	1 (2)	1 (3)	0
Hypertrophy adrenal glands	1 (2)	0	1 (6)
Polycystic kidneys	1 (2)	1 (3)	0
**Skeletal anomalies/others**			
Scoliosis/skeletal abnormalities	3 (6)	2 (7)	1 (6)
Polydactyly/syndactyly	3 (6)	3 (10)	0
Uterine agenesis/aplasia	2 (1)	1 (3)	1 (6)

### Genetic Evaluation

Genetic testing was performed in 67% (31/46) of the fetuses and the majority had a normal karyotype. One foetus was diagnosed with Bardet–Biedl syndrome, one foetus was diagnosed with micro-deletion 22q11, and one child tested positive for asphyxiating thoracic dysplasia 3 (ATD3) or Jeune syndrome post-natally.

### Post-natal Cohort

Overall, 18/29 (62%) fetuses with LAI and 11/17 (65%) fetuses with RAI were live born. The median birth weight was 3,290 g (range 1,950–5,300 g) in the LAI group and 3,178 g (range 2,464–3,567 g) in the RAI group. Median gestational age at delivery was 38 weeks in both the groups (range LAI 32–42 weeks, range RAI 35–40 weeks). A description of clinical data of foetal and neonatal deaths after prenatal diagnosis is shown in Supplementary Table 1.

Follow-ups were available between 1 day and 18 years after birth. At 1 year of age, the overall survival [estimate (95% CI)] was 67% (48; 92%) in patients with LAI and 55% (32; 94%) in patients with RAI. At 5 years of age, the estimates were 67% (48; 92%) in the LAI group and 46% (24; 87%) in the RAI group. Median survival (Q1; Q3) was 11.1 (0.1; 14) years for patients with LAI and 1.3 (0.09; NA) years for patients with RAI. The survival rate of live births with LAI (95% CI) was 67% (0.48; 0.92) at 10 years and 22% (0.04; 1) at 15 years. The survival rate of live births with RAI (95% CI) was 46% (0.24; 0.87) at 10 years and 46% (0.24; 0.87) at 15 years.

**FIGURE 3:**
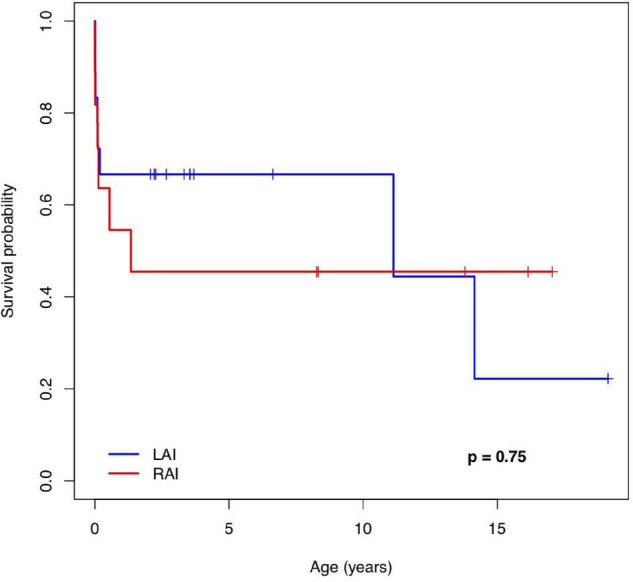
The Kaplan–Meier survival curve of patients with a pre-natal diagnosis of left and right atrial isomerism. LAI, left atrial isomerism; RAI, right atrial isomerism.

Figure 3 shows the Kaplan–Meier survival curve for 29 live-born children with HS. There was no significant difference in the survival rate between the LAI and RAI groups [HR (95% CI): 0.84 (0.29; 2.44), *p* = 0.75]. In addition, there was no difference in the era-specific outcome [HR (95% CI): 1.01 (0.31; 3.37), *p* = 0.98]. Gestational week (GW) at delivery was the only variable with a statistically significant effect on survival [HR (95% CI): 0.62 (0.47; 0.82), *p* = 0.0009]. Detailed information on mid-long-term survivors is given in Supplementary Table 2.

### Discordant Anatomical Patterns Compared to Initial Pre-natal Diagnosis

**FIGURE 4:**
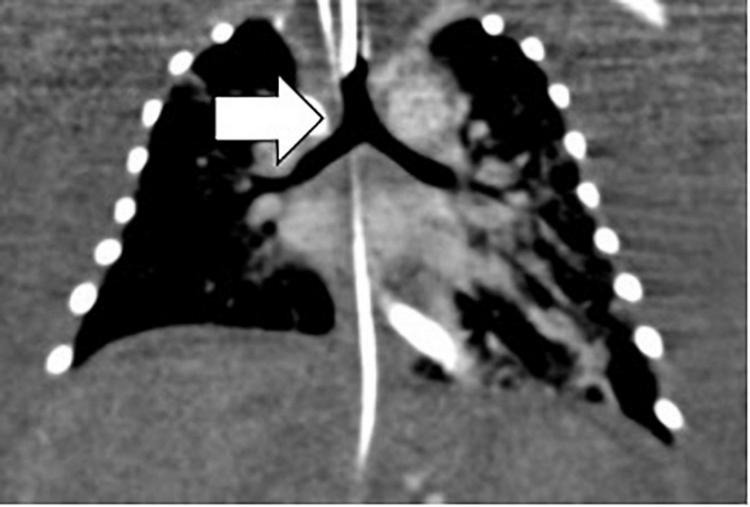
Bronchial tree anatomy and the sub-type of isomerism by neonatal thoracic CT. Arrow white, left isomerism of tracheal bifurcation with bi-lobar lungs.

**FIGURE 5:**
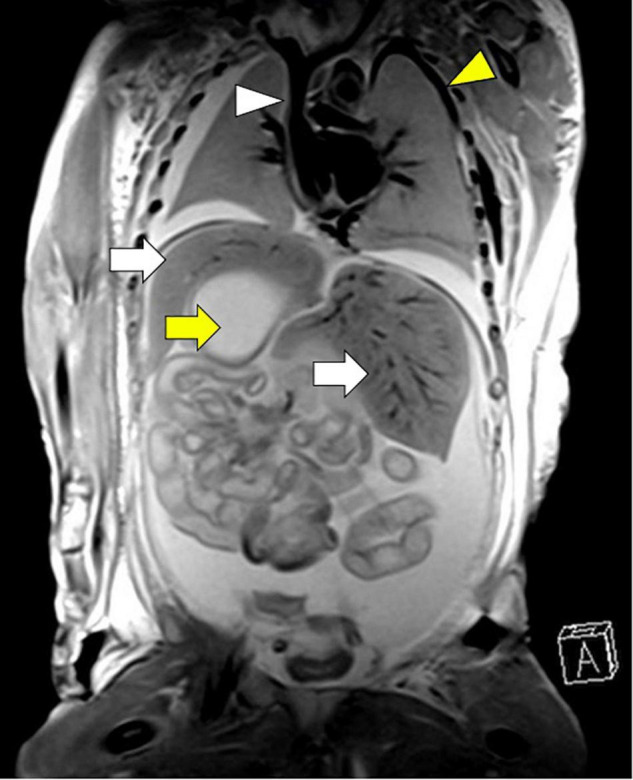
Concordant pattern of broncho-pulmonary branching confirmed by post-mortem MRI in the left atrial isomerism cohort. Arrows: white, bilateral liver; yellow, right-sided stomach. Arrowheads: white, right atrium with insertion of vena cava superior, but without vena cava inferior, yellow, vena azygos supplying blood of the lower body half. Gestational age at MRI is 23 weeks + 4 days.

Investigation of discordant or concordant post-natal patterns after a foetal diagnosis of HS was possible in 11/29 (38%) live-born children. 10/29 (34%) live-born children had a thoracic CT scan post-natally (Figure 4). One foetus had a post-mortem MRI (Figure 5).

Broncho-pulmonary branching: 6/10 (60%) children showed concordant patterns regarding the bronchial tree anatomy and the sub-type of isomerism diagnosed pre-natally and 4/10 (40%) children showed discordant patterns. Three children had post-natally confirmed situs inversus thoracalis, two of which were initially diagnosed with right isomerism and one of which was diagnosed with left isomerism based on the foetal situs view. One child was diagnosed with LAI, but had bronchial anatomy that is typical for RAI. The post-mortem MRI of the foetus showed a concordant pattern of broncho-pulmonary branching with the initial foetal definition of LAI.

Atrial appendage arrangement: 5/10 (50%) children showed a concordant pattern regarding the atrial appendage arrangement and the sub-type of isomerism diagnosed prenatally and 4/10 (40%) children showed discordant patterns. It was not possible to determine the atria on the post-mortem MRI.

Splenic status: In our LAI cohort, there were seven cases with asplenia, 13 cases with polysplenia, and six cases with a regular spleen. In three fetuses, a determination was not feasible. In the RAI cohort, there were 13 cases with asplenia, none with polysplenia, four with regular spleen locations, and one was not determinable.

### Clinical Outcomes in Left Atrial Isomerism

Of the 18 live-born children, three neonatal deaths occurred due to inoperable complex congenital heart disease, hydrops, and/or major extra-cardiac anomalies. Seven children (7/18, 39%) did not require cardiac surgery and are alive and well. Eight (8/18, 44%) children underwent cardiac surgery: primary bi-ventricular repair in 2/8 (25%), initial uni-ventricular palliation with conversion to bi-ventricular circulation in 2/8 (25%), and primary uni-ventricular palliation in 4/8 (50%). There were no survivors in the uni-ventricular palliation group. Reasons for death were extra-cardiac complications (*n* = 3) and septic multi-organ failure (*n* = 1) after a Glenn/Kawashima procedure with the incorporation of the hepatic veins. The bi-ventricular conversion was successful in two children with a late death at 14 years of age in one child. Three (17%) children who had surgery due to extra-cardiac anomalies underwent a Kasai procedure and liver transplantation (*n* = 1), duodenal resection (*n* = 1), or surgery for polydactyly (*n* = 1).

### Clinical Outcomes in Right Atrial Isomerism

Of the 11 live-born children with RAI, comfort care was planned for two children due to inoperable heart disease and extra-cardiac anomalies. Nine children (9/11, 82%) had cardiac surgery. Nine children were initially planned for uni-ventricular repair: Blalock–Taussig (BT) shunt (*n* = 9), Glenn procedure, and subsequent total cavopulmonary connection (TCPC) palliation (*n* = 5). All the children with Fontan completion were alive and well at the time of data collection. Initial uni-ventricular repair with later conversion to bi-ventricular repair was planned in one child with AVSD, a double outlet right ventricle, transposition of the great arteries, pulmonary stenosis, and TAPVD. This child died in the post-operative period after conversion surgery at 7 months. Surgical intervention for extra-cardiac anomalies was performed in one child with duodenal atresia.

## Discussion

Heterotaxy syndrome is defined as an abnormal arrangement of thoracic and abdominal organs often associated with a cardiac defect and major extra-cardiac malformations. In most cases, a multi-disciplinary approach is mandatory to provide good clinical care. The patients discussed in this article emphasise the complexity of managing patients with HS. The present cohort was evaluated with different types of imaging modalities, FE, obstetric US, foetal MRI, and post-natal echocardiography, US and CT scans. In addition, autopsy reports and pm MRI data were available. We have already published our summary on systematic phenotypic characterisation of fetuses with HS using the maximally available prenatal imaging methods (foetal MRI and US). In doing so, we were able to identify a series of extra-cardiac abnormalities, which can help to further specify the sub-type of HS (19). As this entity is rare, peri-natal and post-natal follow-up reports are particularly helpful for clinicians to provide accurate foetal counselling. Our study cohort was evaluated over a period of almost 21 years, which helped us understand the complexity of this entity even more. Even though this study is limited by the retrospective data collection and the small sample size in the outcome group, the present data is valuable for the current discussion on the nomenclature. In the following discussion, a step-wise approach is outlined to provide information for prenatal counselling based on our findings and the current literature. Post-natal nomenclature, definition, and classification (4) remain an area of debate due to the complex nature of this diagnosis (12–14, 31, 32). It has not only been questioned if the terms isomerism and HS can be used as synonyms (3, 12–14, 32, 33), but also if it is appropriate to make a sub-type definition based on the currently used criteria (3, 13), as they might not always be consistent (13). The foetal definition of the sub-types is mainly based on the location of the aorta and IVC in the abdominal situs view and has been used relatively accurately (13, 34). Houyel et al. found different patterns (atrial pectinated muscles and status of the spleen) that were not always concordant with classical LAI and RAI in 44% of fetuses. However, the bronchial status was always concordant (14). In our cohort, 40% displayed a discordant pattern, including broncho-pulmonary branching and atrial appendage anatomy, which are in accordance with the scarce published literature on this particular topic (13, 14). To the best of our knowledge, this is the first study to investigate concordant and discordant patterns in live-born children after a foetal diagnosis of HS. The interpretation of our findings may certainly be biased and, therefore, limited by the fact that only 11 post-natal imaging studies were retrospectively available (10 CT scans, 1 pm MRI), as these were the only children for whom further imaging was necessary for surgical planning.

Nevertheless, we strongly agree with Yim et al. (13) and Houyel et al. (14) that the terms “isomerism” and “heterotaxy” cannot be used synonymously and that each anatomic feature should be described individually rather than categorically.

*In utero* diagnosis of HS is feasible, especially in 733 tertiary care centres (13, 20, 34–36). The rate of TOP (12/46, 26%) in our cohort is comparable to previously reported in a study by Akalin et al. (10), slightly higher than in a study by Escobar-Diaz et al. (20), but lower than the data reported in a study by Vigneswaran et al. (8). Several factors such as gestational age at diagnosis, presence of cardiac and extra-cardiac anomalies, and specific state law regulations might be responsible for the different TOP rates. There were no natural foetal deaths. In LAI, rhythm disturbances with a higher degree of AV block were present in 25%, resulting in a higher proportion of TOP or timely post-natal demise.

With respect to the foetal diagnosis of HS, suspicion commonly arises while examining the foetal heart. The cardiac findings are in accordance with previously published results (8–10, 37). RAI is generally associated with more complex congenital heart diseases with a poorer prognosis (22, 38).

Total anomalous pulmonary venous drainage (TAPVD) was more frequent in RAI than in LAI (53 vs. 7%), as were more complex anomalies with right outflow tract obstruction such as pulmonary stenosis or atresia (82%). Co-arctation was mainly seen in LAI (28%). Importantly, 20% of fetuses with LAI did not have any cardiac defect apart from interruption of the IVC and azygos continuity. In comparison, Gilljam et al. reported 13% (37) and Vigneswaran et al. (8) reported 9% in their cohort. An explanation might be that these fetuses presented with extra-cardiac anomalies and in turn, a detailed foetal echocardiogram was initiated. In conclusion, we would like to stress that a detailed foetal echocardiogram with situs evaluation should be included in the work-up for extra-cardiac anomalies in all the fetuses. Even if the intra-cardiac anomaly is normal, rhythm disorders can occur in this cohort and follow-up is warranted. Due to the presence of two anatomically left atria, the sinus node is missing in fetuses with LAI, thus resulting in rhythm disorders already present *in utero*. Foetal bradycardia was present in 24% and four cases (4/7) developed hydrops, which is comparable to the 24% with bradycardia or AVB reported in a study by Escobar-Diaz et al. with a survival rate of 63% (39). 10% of our cohort needed pacemaker implantation post-natally, which is a lower rate than reported in a study by Baban et al. (1). All of them had sinus rhythm in the foetal period and developed overt rhythm disorders either post-natally or post-surgically. Extra-cardiac anomalies (apart from the situs and spleen) were found in 16/46 (35%) fetuses, which was less frequent than the 62.5% reported in a study by Escobar-Diaz et al. (20), but higher than the 15.8% reported in a study by Gottschalk et al. (40). Escobar-Diaz et al. found a rather high rate of gut-malrotation post-natally, as they run a general screening program for this anomaly at their institution (20). In our study, malrotation of the gut was suspected in foetal MRI (19) or found in autopsy. Overall, 39 mid-line-associated defects were present in 35% of the fetuses, which is in line with the 38% reported in a study by Ticho et al. (18). While additional imaging with foetal MR helps significantly, there is a relatively high percentage of autopsy cases in our study. Non-cardiac malformations were present in 10/14 (71%) TOP. Additional anomalies in post-natal (post-mortem) evaluation (autopsy or post-natal MRI) were found in 18% of these TOP fetuses. This is a common finding in foetal imaging due to the limited resolution in small fetuses.

All the live-born children with asplenia or polysplenia received prophylactic antibiotic treatment according to the guidelines and none had severe recurrent infections requiring hospitalisation. This is in accordance with a study by McGovern et al. where the absence of a spleen was not associated with poor outcomes (41). In contrast, Chiu et al. reported a higher rate of community-acquired severe bacterial infections due to lower memory B cell and immunoglobulin M (IgM) memory B-cell percentages. They note higher mortality compared to other cardiac patients, regardless of the presence of a spleen (42). Chromosomal disorders, such as aneuploidies, complex chromosomal rearrangements, and micro-deletions, have been described in HS, albeit very rarely (43). Our retrospective evaluation spans over 21 years and genetic testing strategies have changed tremendously during this time. The majority only underwent karyotyping and the results are not representative of the whole possible spectrum of chromosomal/genetic abnormalities. Ciliary dysfunction is increasingly recognised in HS. Of the three (3/29, 10%) live-born children tested for cilia dysfunction, one turned out to be pathologic. Nakhleh et al. reported a rather high prevalence (42%) in their cohort (44). A general screening program for ciliopathies in children with HS has only been established in some institutions.

The outcome, which has mostly been reported in relatively small cohorts, is associated with significant morbidity and mortality after the newborn period, both for LAI and RAI (16, 21, 22, 41, 45, 46). Banka et al. reported an overall mortality of 40% with a median follow-up of 1.2 years, 24% of which died of non-cardiac causes. In 36% of 106 children, the reason for death was unknown (16). In the neonatal period, death in our cohort was due to comfort care (*n* = 5). In the first 2 years of life, death was mainly attributable to complex cardiac procedures with extra-cardiac complications (*n* = 7) and after 10 years, it was the result of multi-organ failure after highly complex cardiac surgery (*n* = 2). Mortality is an extremely important topic and nearly always a major concern for parents during foetal counselling. Generally, the prognosis is reported to be worse in RAI than in LAI (9, 20, 22, 38). Long-term survival at 15 years of age was 46% in the RAI group vs. 22% in the LAI group, which is comparable to data published in a study by Vigneswaran et al. (8) and very recently in a study by Akalin et al. (10), but with quite a different pattern. A higher rate of late deaths in the LAI group was seen in children who required complex cardiac procedures. Early deaths did not complicate Fontan palliation and the long-term outcome was favourable for these children. A recent meta-analysis of 848 cases found higher early mortality in patients with HS and completed Fontan palliation compared to the overall Fontan population. Long-term survival, on the contrary, was reported to be acceptable and predictable in this study (47), in accordance with data published in a study by Baban et al. and Azakie et al. who reported reduced mortality rates and better long-term survival rates in patients with HS after Fontan procedure (1, 45). Banka et al. examined the outcome of patients born with HS between 1984 and 2014 (16). Patients were divided into four eras and the long-term outcome was assessed. The outcome remained poor with an overall mortality rate of 40% and did not improve over time. As expected, the greatest risk factors predicting poor outcomes were uni-ventricular circulation and TAPVD. Extra-cardiac morbidity was not reported (16). Therapeutic concepts for the complex cardiac disease have clearly evolved over time, but era-specific outcomes concerning survival in our cohort did not differ from each other. This might in part be due to the small sample size of each cohort.

Predictors for survival were difficult to assess. The sample size is small, which limited the power of the statistical analysis. However, again as expected, gestational weight at delivery turned out to be a statistically significant factor.

### Limitations

This study has several limitations. It presents retrospective data from one tertiary centre comprising only 46 fetuses and 29 live-born children. Substantial data (e.g., for proper classification) are missing, as we included fetuses retrospectively rather than prospectively, making it difficult to follow a uniform protocol. This was especially true for our comparison of the discordant post-natal patterns with the pre-natal diagnosis, where the necessary additional imaging was only available in 11 children. Only fetuses with situs anomalies and cardiac defects pre-natally diagnosed as HS could be included. Therefore, cases with missed cardiac defects or fetuses with cardiac defects and not-detected bronchial isomerism were not enrolled. During the 21-year study period, pre-natal imaging, neonatal management, surgical procedures, and interventional cardiology significantly evolved, which may have influenced the outcome and weakened comparison. In addition, the neurological outcome, psychological development, and psychosocial status of these children were not sufficiently available, even though these factors are now uniformly assessed in most paediatric cardiology centres. The data have been becoming increasingly available, providing important information for improving the quality of prenatal counselling.

## Conclusion

HS is a rare and complex condition with significant morbidity and mortality related to severe cardiac and extra-cardiac manifestations. Accurate pre-natal diagnosis can help identify the fetuses at risk and ensure timely intervention in a multi-disciplinary setting. Further studies are warranted to shed light on the exact sub-type definition in fetuses with HS and the presence of discordant post-natal patterns. The sequential segmental approach of this multi-disciplinary disease is important.

## Data Availability Statement

The original contributions presented in this study are included in the article/Supplementary Material, further inquiries can be directed to the corresponding author.

## Ethics Statement

The studies involving human participants were reviewed and approved by Ethical Committee of the Medical University of Vienna, Austria. Written informed consent to participate in this study was provided by the participants or their legal guardian/next of kin.

## Author Contributions

ES-M, IM-B, and BU designed the study. VJ, AW, GK, EK, BU, MS, and DZ contributed to the design and implementation of the research, to the analysis of the results, and to the writing of the manuscript. ES-M and IM-B wrote the manuscript with input from all authors. IS performed the statistical analysis. All authors contributed to the article and approved the submitted version.

## Conflict of Interest

The authors declare that the research was conducted in the absence of any commercial or financial relationships that could be construed as a potential conflict of interest.

## Publisher’s Note

All claims expressed in this article are solely those of the authors and do not necessarily represent those of their affiliated organizations, or those of the publisher, the editors and the reviewers. Any product that may be evaluated in this article, or claim that may be made by its manufacturer, is not guaranteed or endorsed by the publisher.
