# Supplementary material for: Discordant Post-natal Patterns in Fetuses With Heterotaxy Syndrome: A Retrospective Single-Centre Series on Outcome After Fetal Diagnosis
Source: Front Pediatr. 2022 Jul 14;10:908505. doi: 10.3389/fped.2022.908505 (PMC9329514; doi:10.3389/fped.2022.908505)
Supplement: Supplementary file 1 [file Table_1.DOCX]

**Supplement Table 1 : Clinical data of fetal and neonatal deaths after prenatal diagnosis of heterotaxy, sorted by left atrial isomerism (LAI; n = 14) or right atrial isomerism (RAI; n = 8), according to length of follow up.**

| **GA at diagnosis (weeks)** | **Sex** | **Abnormal cardiovascular finding** | **Genetic workup** | **Non-cardiac findings according to imaging and autopsy** | **Spleen** | **GA and birth-weight at delivery** | **Outcome (GA and weight at autopsy)** |
| --- | --- | --- | --- | --- | --- | --- | --- |
| **LAI** |  |  |  |  |  |  |  |
| 12+5 | m | Single ventricle, AVSD, DORV, CoA; (AVB) | Normal karyotype | SA, NT 5.1mm, dilated intestines, skin edema, gall bladder aplasia, duodenal atresia | Polysplenia |  | TOP (18+1;200gr) |
| 13+5 | m | Common atrium, Hypoplastic RV,AVSD, TAPVD, Bilateral SVC; (2nd-3rd degree AVB) | Normal karyotype | SA, NT 3.3mm, craniofacial dysmorphy, hyperechogenic intestines and lungs | Polysplenia |  | TOP (15+3;110gr) |
| 15+5 | f | Hypoplastic RV, VSD, PA; (SR) | Normal karyotype | SA, craniofacial dysmorphy, hygroma colli, cleft palate, anal atresia, uterine aplasia, urethral obstruction | Polysplenia |  | TOP (16+5; 105gr) |
| 16+3 | f | CoA, (AVB) | Normal karyotype | SA, hydrops, NT 6.1mm, skin edema, hyperechogenic intestines, hydrothorax | Polysplenia |  | TOP (17+0; 150gr) |
| 20+3 | f | AVSD, DORV, PA, Bilateral SVC; (SR) | Normal karyotype | SA, cleft lip palate, craniofacial dysmorphy, poly/syndactyly, gallbladder aplasia, short intestines, short femur | Small spleen |  | TOP (22+4; 475gr) |
| 20+6 | f | TGA, VSD, PA (SR) | Normal karyotype | SA | Asplenia |  | TOP (21+6; 448gr) |
| 23+0 | f | Common atrium, Single ventricle, PS (SR) | Normal karyotype | SA, DWM, omphalocele, malrotation | Regular (right) |  | TOP (23+4; 612gr) |
| 20+6 | m | AVSD, DOLV, TGA, Bilateral SVC; (2nd to 3rd degree AVB) | none | SA, malrotation, hydrops, polyhydramnios | Polysplenia | 34+1, 5300gr | Hydropic, died 10 min pp |
| 30+2 | m | Single ventricle, AVSD, AS, CoA PAPVD, bilateral SVC (SR) | none | SI, craniofracial dysmorphy, ventriculomegaly, lung hypoplasia, bilobular lungs, Potter-Sequence, bilateral kidney agenesis | Polysplenia | 32+1, 1950gr | Died 4h pp |
| 37+0 | m | Single ventricle, AVSD, TGA, CoA, TAPVD, bilateral SVC, I-IVC (SR) | none | SA | Asplenia | 40+6, 3190gr | Inoperable, died 1st week |
| 14+0 | n.k. | I-IVC; (SR) | none | SA | Asplenia |  | LTFU |
| 18+4 | n.k. | I-IVC; (SR) | none | SA | n.k. |  | LTFU |
| 26+4 | n.k. | AVSD, DORV, MGA, PA, I-IVC (SR) | none | SI, dilated intestines | n.k. |  | LTFU |
| 27+1 | n.k. | AVSD, AS, CoA (AVB) | none | SA, hydrops, macrosomia | n.k. |  | LTFU |
| **RAI** |  |  |  |  |  |  |  |
| 17+2 | f | TGA,PA,VSD (SR) | Normal karyotype | SI, skin edema, hematoma of the liver | Regular (left) |  | TOP (18+6;240gr) |
| 17+5 | f | Hypoplastic RV, PA (SR) | Normal karyotype | SA, hydrocephalus, cerebellar hypoplasia, rhombencephalosynapsis, kidney agenesis right, uterine agenesis, dysplasia of the right thumb, scoliosis, lung hypoplasia, monolobular lung right | Asplenia |  | TOP (18+6; 216gr) |
| 18+5 | m | Hypoplastic LV, TGA, VSD, PS, AA, TAPVD (SR) | none | partial SI, gallbladder left, bilateral trilobular lungs | Regular (left) |  | TOP (20+0; 420gr) |
| 21+6 | m | Single ventricle, AVSD, TGA, PS, TAPVD (SR) | Normal karyotype | SA, bilateral trilobular lungs, hypertrophy of the adrenal glands | Asplenia |  | TOP (22+6; 640gr) |
| 23+0 | m | Hypoplastic LV, TAPVD, AS, Bilateral SVC (SR) | Normal karyotype | SA, trilobular lungs bilateral, malrotation, duplex kidney right | Asplenia |  | TOP (25+1; 820gr) |
| 20+6 | m | Single ventricle, AVSD, AA, CoA (SR) | Normal karyotype | SA, skin edema, bilateral hydronephrosis | Asplenia | 39+5, 2464gr | Died 1st week |
| 30+6 | m | Hypoplastic LV, AVSD, DORV, TGA, PA, TAPVD (SR) | Normal karyotype | SA, ventriculomegaly, retrognathia, dilated intestines, malrotation | Asplenia | 39+5, 2700gr | Inoperable, died 1st week |
| 17+6 | n.k. | AVSD, TGA, PA, Bilateral SVC (SR) | Normal karyotype | SI | n.k. |  | LTFU |

AA, aortic atresia; AS, aortic stenosis; ASD, atrial septal defect; AVSD, atrio-ventricular septal defect; AP shunt, aortopulmonary Shunt; AVB, atrioventricular block; CoA, BT shunt, Blalock-Taussig shunt; coarctation of the aorta; CS, coronary sinus; DOLV, double-outlet left ventricle; DORV, double-outlet right ventricle; GA, gestational age; hypoplastic LV, hypoplastic left ventricle; hypoplastic RV, hypoplastic right ventricle; I-IVC, interrupted inferior vena cava; LAI, left atrial isomerism; LTFU, lost to follow up; NT, nuchal translucency; PA, pulmonary atresia; PAPVD, partially anomalous pulmonary venous drainage; PM, pacemaker; PS, pulmonary artery stenosis; RA, right atrium; RAI, right atrial isomerism; SA, situs abnormality; SI, situs inversus abdominalis; SR, sinus rhythm; SVC, superior vena cava; TAPVD, total anomalous pulmonary venous drainage; TGA, transposition of the great arteries; TOP, termination of pregnancy; VSD, ventricular septal defect;
