# Supplementary material for: Discordant Post-natal Patterns in Fetuses With Heterotaxy Syndrome: A Retrospective Single-Centre Series on Outcome After Fetal Diagnosis
Source: Front Pediatr. 2022 Jul 14;10:908505. doi: 10.3389/fped.2022.908505 (PMC9329514; doi:10.3389/fped.2022.908505)
Supplement: Supplementary file 2 [file Table_2.DOCX]

**Supplement Table 2. Mid-long-time survivors after prenatal diagnosis of heterotaxy, sorted by left atrial isomerism (LAI; n = 15) or right atrial isomerism (RAI; n = 9), according to length of follow up.**

| **GA at diagnosis (weeks)** | **Sex** | **Abnormal cardiovascular findings** | **Genetic workup** | **Non- cardiac findings according to imaging** | **Spleen** | **GA and birth-weight at delivery** | **Postpartum interventions** | **Outcome (Follow up)** |
| --- | --- | --- | --- | --- | --- | --- | --- | --- |
| **LAI** |  |  |  |  |  |  |  |  |
| 22+0 | f | Hypoplastic LV, AS, CoA, I-IVC (SR) | Normal karyotype | SI, growth restriction | Polysplenia | 38+4,3465g | Norwood procedure, VAD support | died at 5 weeks |
| 23+0 | f | AVSD, CoA, PAPVD, I-IVC (2nd-3rd degree AVB) | Normal karyotype | SA, malrotation, double bubble | Polysplenia | 37+0, 3400g | PM, bilateral PA banding, compartment syndrome due to thrombosis femoral vein | died at 2 months |
| 33+4 | m | Hypoplastic LV, TGA, VSD, PS, bilateral SVC, I-IVC (SR) | none | SA, craniofacial dysmorphy, hydronephrosis left | Asplenia | 37+5, 2975g | BT Shunt | died at 2 months |
| 24+0 | f | I-IVC (fetal SR, postnatal 1st degree AVB) | Normal karyotype | SI | Regular (right) | 40+0,2980g |  | A&W (6 years) |
| 28+3 | m | I-IVC (2nd degree AVB) | none | SA, malrotation, volvulus | Polysplenia | 36+6, 2910g | Duodenal resection | A&W (2 years) |
| 23+4 | m | I-IVC (SR) | Bardet-Biedel-Syndrome (Type 2) | SA, polydactyly, polycystic kidneys | Regular (right) | 41+2, 3700g | Surgery of polydactyly | A&W (2 years) |
| 19+3 | f | Hypoplastic LV, AS, CoA, I-IVC (SR) | ATD3 ciliopathy (Jeune syndrome) | SA, malrotation, short-rib polydactyly syndrome, skeletal dysplasia | Polysplenia | 39+0, 2570g | Norwood procedure, conversion to biventricular repair | A&W (3 years) |
| 20+3 | m | I-IVC (SR) | Normal karyotype | SA,ventricular asymmetry | Asplenia | 39+5, 3290g |  | A&W (3 years) |
| 25+0 | f | DORV, PAPVD (SR) | Normal NIPT | SA | Regular (right) | 40+3, 4050g | Rastelli type VSD closure | A&W (3 years) |
| 28+4 | f | VSD, I-IVC (SR) | DiGeorge-syndrome | SA, malrotation | Asplenia | 39+3, 3290g |  | A&W (3 years) |
| 24+0 | f | I-IVC (SR) | Normal karyotype | SA,PCD | Polysplenia | 41+6, 3340g |  | A&W (2 years) |
| 20+6 | m | Hypoplastic RV, DORV, PS, PAPVD, bilateral SVC, I-IVC (fetal SR, postnatal bradycardia) | Normal karyotype | SA, malrotation | Polysplenia | 37+3, 3460g | BT Shunt, AP Shunt, Glenn/Kawashima procedure, incorporation of the hepatic veins, PM | died at 11 years |
| 20+0 | f | Hypoplastic RV, DORV, PAPVD, bilateral SVC, I-IVC (fetal SR, postnatal bradycardia) | Normal karyotype | SA,malrotation, duplex kidney | Polysplenia | 38+1, 3800g | BT Shunt, AP Shunt, unroofing CS, PM, Glenn procedure, conversion surgery (ASD+VSD closure, Inspiris valve in Pulmonary artery position, connection of systemic veins to RA) | died at 14 years |
| 20+0 | m | I-IVC; (SR) | none | SA | Regular | n.k. |  | A&W (18 years) |
| 33+2 | f | Double chambered RV, VSD, bilateral SVC, I-IVC (SR) | none | SA, gallbladder aplasia, biliary atresia, malrotation, growth restricition | Asplenia | 37+6, 2740g | RV myectomy, VSD closure, Kasai procedure, liver transplantation | A&W (12 months) |
| **RAI** |  |  |  |  |  |  |  |  |
| 25+3 | m | Hypoplastic RV, AVSD, TAPVD, Bilateral SVC (SR) | Normal karyotype | SA, hydronephrosis right | Asplenia | 38+0, n.k. | BT Shunt | died at 5 weeks |
| 36+0 | m | Hypoplastic RV, TGA, PA, TAPVD, single left SVC (SR) | none | SA | Asplenia | 36+1, 3300g | BT Shunt | died at 5 weeks |
| 21+5 | m | Common atrium, AVSD, DORV, TGA, PS, TAPVD (SR) | Normal karyotype | SA, ventricular asymmetry, duodenal atresia, cleft palate | Asplenia | 38+0, 2560g | BT Shunt, duodenal surgery, corrective surgery (RVPA conduit, LeCompte, Shunt Ligation) | died at 7 months |
| 29+6 | m | Common atrium, AVSD, DORV, TGA, PS, PAPVD (SR) | Normal karyotype | SA, subdural hygroma, delayed mylelinization | Asplenia | 35+1, 2480g | BT Shunt | died at 15 months |
| 20+5 | m | Single ventricle, PA, PAPVD, bilateral SVC (SR) | none | SI | Regular (right) | 40+0, 3545g | AP Shunt, Glenn procedure, TCPC | A&W (8 years) |
| 21+3 | m | Common atrium, AVSD, TAC TAPVD, I-IVC (SR) | Normal karyotype | SA | Asplenia | 39+2, 3160g | BT Shunt, Glenn procedure, TCPC | A&W (8 years) |
| 21+6 | f | Single ventricle, TGA, PA, PAPVD (fetal SR, postnatal bradycardia) | Normal karyotype | SA | Regular (right) | 40+2, 3263g | BT Shunt, Glenn procedure, TCPC, PM | A&W (13 years) |
| 24+0 | m | Single ventricle, AVSD, PA, bilateral SVC (SR) | none | SI, hydrocephalus, T-cell aplasia, malrotation | Asplenia | 38+4, 3567g | BT Shunt, Glenn procedure, TCPC | A&W (16 years) |
| 35+1 | m | Single ventricle, common atrium, AVSD, TGA, PS, TAPVD (SR) | Normal karyotype | SA | Asplenia | 40+1, 3195g | Interventional ballon valvuloplasty, BT Shunt, Glenn procedure, TCPC, mechanical MV | A&W (17 years) |

A&W, alive and well; AS, aortic stenosis; ASD, atrial septal defect; AP shunt, aortopulmonary Shunt; AVB, atrioventricular block; AVSD, atrio-ventricular septal defect; BT shunt, Blalock-Taussig shunt; CoA, coarctation of the aorta; CS, coronary sinus; DOLV, double-outlet left ventricle; DORV, double-outlet right ventricle; GA, gestational age; Hypoplastic LV, hypoplastic left ventricle; Hypoplastic RV, hypoplastic right ventricle; I-IVC, interrupted inferior vena cava; LAI, left atrial isomerism; MV, mitral valve; NIPT, noninvasive prenatal testing; PA, pulmonary atresia; PA Banding, pulmonary artery banding; PAPVD, partially anomalous pulmonary venous drainage; PM, pacemaker; PCD, primary ciliary dyskinesia; PS, pulmonary artery stenosis; RAI, right atrial isomerism; RA, right atrium; RV, right ventricle; SA, situs abnormality; RVPA conduit, right ventricle to pulmonary artery conduit; SR, sinus rhythm; SVC, superior vena cava; TAC, truncus arteriosus communis; TAPVD, totally anomalous pulmonary venous drainage; TCPC, total cavo-pulmonary connection; TGA, transposition of the great arteries; VSD, ventricular septal defect;
